# Individualised tobacco affordability in the UK 2002–2014: findings from the International Tobacco Control Policy Evaluation Project

**DOI:** 10.1136/tobaccocontrol-2017-054027

**Published:** 2018-07-23

**Authors:** Timea R Partos, J Robert Branston, Rosemary Hiscock, Anna B Gilmore, Ann McNeill

**Affiliations:** 1 Addictions Department, King’s College London, London, UK; 2 UK Centre for Tobacco & Alcohol Studies (UKCTAS), Nottingham, UK; 3 School of Management, University of Bath, Bath, UK; 4 Department for Health, University of Bath, Bath, UK

**Keywords:** taxation, price, hand-rolled/ryo tobacco, economics, disparities

## Abstract

**Objective:**

The existing measures of tobacco affordability (smokers' purchasing power for tobacco) use national estimates of income and average cigarette prices, and exclude roll-your-own (RYO) tobacco. This study developed an individualised measure of tobacco affordability using smokers' own incomes and factory-made (FM) or RYO tobacco purchase prices, and explored how it was impacted by taxation changes, individual characteristics and purchase patterns.

**Design:**

Cross-sectional survey data collated from 10 waves of a longitudinal cohort study.

**Data sources:**

Adult smokers (n=4062) from the International Tobacco Control Policy Evaluation Project United Kingdom (UK), surveyed between 2002 and 2014, providing 8943 observations over 10 surveys.

**Analysis:**

Affordability was calculated as the percentage of annual income remaining with the individuals after their annual tobacco expenditure. Multilevel linear regression models were used with affordability as the outcome using time, sex, age, geographical region, ethnicity, education, nicotine dependence and tobacco purchase source as the predictor variables.

**Results:**

Affordability of FM cigarettes decreased significantly from 91.5% (±95% CI: 91.0% to 91.9%) in 2002 to 87.8% (87.0% to 88.5%) in 2014; and RYO from 96.3% (95.7% to 96.9%) in 2006 to 93.7% (93.0% to 94.4%) in 2014. Affordability was significantly lower for FM than RYO. Year-on-year decreases were not statistically significant. Tobacco was more affordable for males, those with higher education, less dependent smokers and those purchasing from non-store (potentially illicit) or non-UK sources.

**Conclusions:**

An individualised measure of tobacco affordability provided useful insights on the impact of tobacco taxes, social inequalities and purchase patterns in the UK. Although tobacco became less affordable, the annual rate of decline was low, suggesting annual tax rises were not large enough.

## Introduction

Raising cigarette prices through tobacco taxation is one of the most effective and socially equitable tobacco control measures.^1–4^ However, the impact of price rises is modified by inflation rates and changes in incomes.^5^ Therefore, affordability (an indicator of smokers' purchasing power for tobacco with respect to both income and tobacco prices) needs to be understood to measure the impact of tobacco taxes.^6^ More affordable cigarettes lead to increases in consumption.^7^ Several measures of tobacco affordability have been developed to assess affordability. These measures have been standardised to enable affordability to be measured within countries over time, and to allow for between-country comparisons.

Existing tobacco affordability measures include the ‘Big Mac index’ representing the number of cigarettes purchased for the price of one McDonald’s Big Mac hamburger^8^; the ‘minutes of labour’ (MoL) needed to purchase a pack of 20 Marlboro cigarettes or an equivalent local brand^9^; the ‘relative income price’ (RIP) representing the percentage of per capita gross domestic product (GDP) required to purchase 100 packs of cigarettes (Marlboro or local brand)^5^; and the ‘cigarette price daily income ratio’ (CPDIR), which divides the price of one pack of cigarettes (Marlboro or local brand) by daily income.^10^ These measures have different strengths and weaknesses and their merits, particularly when compared across high-income, middle-income and low-income countries, have been discussed elsewhere.^11^

The main drawback of these ‘aggregate’ measures is their reliance on average cigarette prices typically derived from only a handful of brands, and on average national estimates of incomes. This can be problematic given the wide income inequalities observed within many countries. To some extent, aggregate measures can capture the range of prices between different factory-made (FM) tobacco brands by using the cheapest local brand as a comparison.^5 7^ However, this still does not fully account for the widening range of difference in prices between different tobacco products (such as FM and roll-your-own (RYO) tobacco) and the numerous strategies smokers can adopt to minimise costs, such as buying in bulk and purchasing from cheaper sources.^12 13^ One study overcame the problem of average prices by using smokers' own reported prices for their most recent tobacco purchase; however, their income measure was still based on per capita GDP.^14^ Furthermore, none of the measures of tobacco affordability to date have included RYO tobacco, a much cheaper alternative to FM cigarettes.^13 15 16^ As smoking is concentrated in more disadvantaged groups in countries with a mature smoking epidemic, using individualised income has advantages over per capita GDP as it captures differences in the distribution of income across the smoker population and also enables a more fine-grained analysis of differences in affordability across socioeconomic groups. While some studies have used the UBS Survey of Earnings survey to indicate national income as it captures income across several different professional groups, the UBS survey is not designed to be representative of earnings across a country and does not cover unskilled work or unemployed people.

Smokers purchasing cheaper tobacco can weaken the relationship between aggregate affordability measures and tobacco consumption. For example, a study of Thai smokers found no significant change in affordability or overall cigarette consumption despite price rises, yet when examining consumption within separate price tiers, a significant decrease was observed in consumption in the upper and middle tiers, which was offset by an increase of consumption in the lowest tier.^17^ Existing aggregate measures of affordability can also limit their estimates of price to fully taxed sources; however, purchasing from low taxed (eg, duty free, cross-border) or untaxed (illicit) sources can also influence affordability.^18–20^ Examining how individual choices and demographics impact tobacco affordability has seldom been a focus of previous research, but assessing them would enable an individualised measure of affordability, which will be complementary to aggregate measures.

The present study aims to examine tobacco affordability in the United Kingdom between 2002 and 2014, while addressing the gaps in the literature outlined above. The UK levies one of the highest tobacco taxes globally, besides having strong tobacco control policies alongside.^21^ Multicountry comparison studies indicate that although cigarettes in the UK have become less affordable since the 1990s, the rate of decline in affordability might be slowing. Between 1991 and 2002, the annual decrease in MoL tobacco affordability was around 5.5%,^9^ but only around 2%–3% between 2003 and 2009.^22^ Similarly, estimates using the RIP indicated an annual decrease in affordability of around 2.5%–3% between 1990 and 2001,^5^ but only 1%–2% between 2004 and 2010.^6^ No studies have examined tobacco affordability in the UK since 2010. Yet, since 2010, the UK has also seen a significant widening of the price range between the cheapest and most expensive tobacco products^12 13^ and an influx of cheaper tobacco brands.^12^ Economic and policy changes have also occurred during this time. Between 2002 and 2008, UK tobacco taxes increased at the rate of inflation, whereas from 2009 to 2014, they were typically around 2% above inflation, with a high of 5% in 2012.^23^ Furthermore, to comply with the European Commission directive 2010/12/EU,^24^ in 2011, the UK began to use the weighted average price (WAP) instead of the most popular price category (MPPC) to calculate tobacco taxes, and also implemented a large increase in the tax on RYO relative to FM cigarettes.^25^ Starting in 2008, the UK also experienced an economic recession.

This study will develop an individualised measure of tobacco affordability, based on smokers' own reported incomes and tobacco purchase prices. Unlike previous research, we will also include RYO tobacco in our analyses. In addition to looking at the change in affordability over time, we will explore the impact of individual differences such as demographics, dependence, tobacco format (FM or RYO) and purchase source (taxed versus low or untaxed). The usefulness of the individualised measure is, first, that it is more representative because it will capture what people are actually spending given that they may be buying cheaper brands, or using cheaper sources, rather than an aggregate measure based on a few brands only and national income estimates. Second, it is more meaningful because it calculates the affordability relative to actual incomes. So it paints a better picture to policy-makers and service providers (for example) about the actual magnitude of the financial burden of smoking for different subgroups. Our findings will therefore help to inform future policy decision-making on tobacco pricing in the UK and possibly elsewhere. However, we note that aggregate measures of affordability have a clear implication, such that when affordability of tobacco changes, for example because of a tobacco price increase, the demand for tobacco decreases. Our individualised measure of affordability depends on *relative* tobacco expenditures, and therefore price increases will not necessarily translate into decreases in demand, and changes in individualised affordability are instead partly a consequence of changes in demand.

## Methods

### Participants

Participants were from the UK arm of the International Tobacco Control (ITC) Policy Evaluation Project, a cohort survey of adult (aged 18 years or over) smokers with replenishment. Ten surveys took place between 2002 and 2014. Surveys are administered via computer-aided telephone interview or online (from 2008 onwards), with stratified random sampling to be representative of the national distributions of age, sex and geographical region. Detailed information about ITC methodology is published elsewhere.^26 27^ We excluded nondaily smokers (n=394), smokers of both FM cigarettes and RYO tobacco throughout (n=636) and exclusive RYO smokers from the first four surveys (n=420) because some questions needed to calculate affordability were not asked. We also excluded invalid responses on tobacco price (n=186, see below), the top and bottom 1 percentile of responses on the affordability variable to minimise outliers (n=480, of which 94% comprised improbable responses such as spending none or over 100% of income on tobacco), and anyone with missing data on the included covariates (n=58). The final sample of 4062 current daily smokers provided 8943 observations over the 10 surveys (average 2.2 observations per individual).

### Measures

#### Affordability

The individualised affordability measure developed in this study was calculated as the percentage of a smoker’s annual gross income remaining after subtracting their annual spend on tobacco (see equation 1), such that higher values represented more affordable tobacco.   Values could theoretically range between 0% and 100%. However, after excluding outliers, affordability in the sample ranged between 35.3% and 99.9%.

(1) $$Individualised\;Affordability\;=\left(\frac{Income\;-\;AnnualTobaccoSpend}{Income}\right)\times 100\%$$

An aggregate measure of affordability, based on average tobacco prices and national estimates of income, was also calculated for comparison. We adapted the methodology for calculating Blecher and Walbeek’s RIP^5^ which is the percentage of per-capita GDP required to purchase 100 packs of 20 FM cigarettes (2000 cigarettes). To make values comparable in magnitude and direction to our measure, we made two adjustments. First, we tripled the number of cigarettes (to 6000 cigarettes or 300 packs of 20) to correspond more closely to the average number of cigarettes smoked per year by our sample, which was 6074 (SD=2913). Second, we inverted the equation so that like our own measure, higher values would indicate more affordable cigarettes. Equation 2 presents the formula for Consumer Price Index (CPI this aggregate affordability measure. UK cigarette prices (FM only) were based on the MPPC from 2002 to 2010 or the WAP from 2011 to 2014, as these are the data published by the European Commission^28^ and on which UK tobacco taxes are based. Cigarette prices and yearly GDP figures were adjusted for inflation to 2014 values using the Consumer Price Index (CPI). GDP and CPI data were obtained from the Office of National Statistics (ONS).^29 30^

(2) $$Aggregrate\;Affordability\;=\left(\frac{GDP\;-TobaccoSpend_{6000\;cigarettes}}{GDP}\right)\times 100\%$$

#### Income

Gross annual household income was reported in ranges (£0–£6499; £6500–£15000; £15 001–£30000; £30 001–£40 000; £40 001–£50 000; £50 001–£65 000; £65 001–£95 000; or £95 001 and higher). To calculate affordability, we took the mid-point of each range and £95 001 for the highest value. Incomes were adjusted to 2014 values using CPI data. Participants also reported their household composition, which was used to derive ‘equivalised’ annual income (adjusted for household composition) using the modified Organisation for Economic Co-operation and Development (mOECD) scale.^31^ Equivalisation weights were modified slightly because children’s ages in the ITC questionnaire were stratified somewhat differently to the mOECD strata (further details are available from the corresponding author). Due to the complexities and slight deviations from the published methodologies involved in equivalising income, we ran sensitivity analyses using a version of income that was not equivalised for household composition. The results of these analyses did not deviate substantially from the results presented using equivalised income and did not alter the conclusions drawn from the data (data not shown).

#### Annual tobacco spend

Participants reported the format of their last tobacco purchase (FM cigarettes by the pack, by the carton or RYO tobacco), including the number of packs, cartons or pouches, and how many packs per carton, cigarettes per pack or grams of tobacco per pouch. The purchase price was also reported. RYO users were also asked how many days a pouch of this weight would usually last, and the number of cigarettes they smoked per day (CPD). This information was used to derive the ‘price per stick’ separately for FM and RYO users.

These calculations for price per stick were adapted from a previous study using this dataset,^13^ and the same exclusion criteria for improbable responses were adopted here. Annual tobacco spend was then calculated by multiplying the price per stick by CPD and by 365. We felt it reasonable to extrapolate annual expenditure from participants' most recent purchase, as the large majority of our sample (92.2% of FM and 95.4% of RYO users) indicated their most recent purchase to be their usual brand.

#### Time (tobacco tax year)

Each ITC survey period spanned a number of months. We assigned participants to the appropriate ‘tobacco tax year’ corresponding to the timing of their response relative to when tobacco tax changes were implemented (March or April each year). No ITC survey data were collected in the 2009 or 2011 tobacco tax years.

#### Demographics

Demographic variables were sex, age, UK geographical region of residence, ethnicity (white or non-white) and highest level of education attained (low=secondary school/vocational level 3 or less; moderate=some college or university but no degree and high=completed university or postgraduate degree). Importantly, level of education served as an indicator of socioeconomic differences, as we could not include income as a covariate because it was used to derive the affordability measure itself.

#### Nicotine dependence

The time to first cigarette (TTFC) after waking was used to indicate dependence, stratified to within 5 min (most dependent); 6 to 30 min; 31 to 60 min; and after 60 min (least dependent).

#### Purchase source

We classified the reported source of participants' last tobacco purchase into two categories using criteria detailed elsewhere.^13^ 1. UK store-based sources (eg, supermarkets, pubs, tobacconists) represented easily accessible and widely used sources that were highly likely to be legal sales. 2. Non-UK/non-store sources (eg, duty free, informal sellers, friends) represented a concerted effort to obtain cheap (potentially including illicit) tobacco.

### Analyses

A basic descriptive comparison of individualised and aggregate affordability was achieved by calculating the changes over time for both the measures. We also computed the changes over time of the constituents of affordability (income and tobacco price) to examine their relative contributions.

To investigate changes in individualised affordability over time, and the associations with individual differences we used multilevel linear random effects regression analyses with maximum likelihood estimation, clustered over individuals. The clustering controlled for correlations between multiple observations provided by the same individual at different surveys. Affordability of FM cigarettes (2002–2014) was analysed separately from RYO tobacco (2006–2014). The dependent variable was our individualised measure of affordability. The independent variables were tobacco tax year (we used the 2002 tobacco tax year as reference, and tested for linear trends, and also conducted reverse adjacent contrasts, which indicated whether each successive period from one survey to the next resulted in a change in affordability that was statistically significant), sex, age and age squared (to test for nonlinear associations with age), geographical region, ethnicity, education, TTFC and purchase source. A random-effect rather than fixed-effect regression model was chosen because of the emphasis on population-level effects rather than cluster-level effects of random effects modelling, its ability to handle small clusters and clusters of one such as was present in our sample, and the ability to model the effects of time-invariant variables such as sex and ethnicity on the outcome. It should also be noted that we recognise the importance of tax changes to affordability. Unfortunately, however, tax changes completely overlapped with the  time variable in our dataset and was therefore a confound that could not be included in our regression model.

Three regression models were computed. Model 1 regressed affordability separately on each independent variable, unadjusted for any other covariates. These univariate analyses indicated if there were any simple associations between each of our independent variables and affordability. Model 2 was fully adjusted for all independent variables concurrently. This indicated which of our independent variables made a significant contribution to predicting affordability, even after controlling for all other included variables. Model 3 repeated model 2 but excluded participants purchasing from non-UK/non-store sources to observe changes in affordability only among sources where full duties were likely to have been paid. Note that neither income nor CPD were included as covariates as these variables were used to derive the affordability measure itself.

A sensitivity analysis was also conducted to examine how smokers changed their tobacco consumption (CPD) over the survey period. This assessed whether any observed changes in affordability were due to changes in tobacco consumption. This analysis regressed CPD onto all the independent variables included in model 2.

## Results

Sample characteristics are presented in table 1. The majority were white, had low to moderate education, smoked their first cigarette within 30 min of waking and purchased tobacco predominantly from UK store-based sources. The mean age was 48 years (*SD*=14), and there were slightly more females (58%) than males. The majority of FM smokers were female (62%), whereas the majority of RYO smokers were male (63%). Although there were some differences in geographical region of residence, the FM and RYO groups were comparable in age, level of education and TTFC. Consistent with previous research,^13 19^ RYO smokers were somewhat more likely to purchase tobacco from non-UK/non-store sources.

**Table 1:** Sample characteristics for the combined sample, and separately by tobacco format: factory made (FM) cigarettes or roll-your-own (RYO) tobacco

	Combined sample		FM smokers		RYO smokers	
	obs	%	obs	%	obs	%
Total observations	8943	100.0	7475	100.0	1468	100.00
Individualised Affordability (%)						
Mean and SD	91.4	9.7	90.6	10.1	95.5	5.9
Annual Income (£)						
Mean and SD	29 347	21 832	30 277	22 464	24 608	17 534
Price per cigarette (£)						
Mean and SD	0.265	0.080	0.273	0.075	0.227	0.092
Cigarettes per day						
Mean and SD	16.8	8.2	16.7	8.1	17.0	9.1
Sex (%)						
Female	5196	58.1	4647	62.2	549	37.4
Male	3747	41.9	2828	37.8	919	62.6
Age						
Mean and SD	49	14.2	48	14.4	50	12.9
Region						
London	1094	12.2	964	12.9	130	8.9
Yorkshire and The Humber	729	8.2	645	8.6	84	5.7
East Midlands	634	7.1	513	6.9	121	8.2
Eastern	753	8.4	601	8.0	152	10.4
North East	393	4.4	340	4.6	53	3.6
South East	1139	12.7	930	12.4	209	14.2
South West	657	7.6	478	6.4	197	13.4
West Midlands	797	8.9	679	9.1	118	8.0
North West	951	10.6	819	11.0	132	9.0
Wales	479	5.4	368	4.9	111	7.6
Scotland	1009	11.3	882	11.8	127	8.7
Northern Ireland	290	3.2	256	3.4	34	2.3
Ethnicity						
White	8534	95.4	7094	94.9	1440	98.1
Not white	409	4.6	381	5.1	28	1.9
Education						
Low	5236	58.6	4389	58.7	847	57.7
Moderate	2380	26.6	1998	26.7	382	26.0
High	1327	14.8	1088	14.6	239	16.3
Time to first cigarette (TTFC)						
Least addicted (over 60 min)	1218	13.6	1066	14.3	152	10.4
31–60 min	1879	21.0	1593	21.3	286	19.5
6–30 min	4407	49.3	3667	49.1	740	50.4
Most addicted (within 5 min)	1439	16.1	1149	15.4	290	19.8
Purchase source						
UK store-based	7495	83.8	6439	86.1	1056	71.9
Non-UK/non-store	1448	16.2	1036	13.9	412	28.1

Total  n=4062,  observations=8943.

### Affordability over time

**Figure 1:**
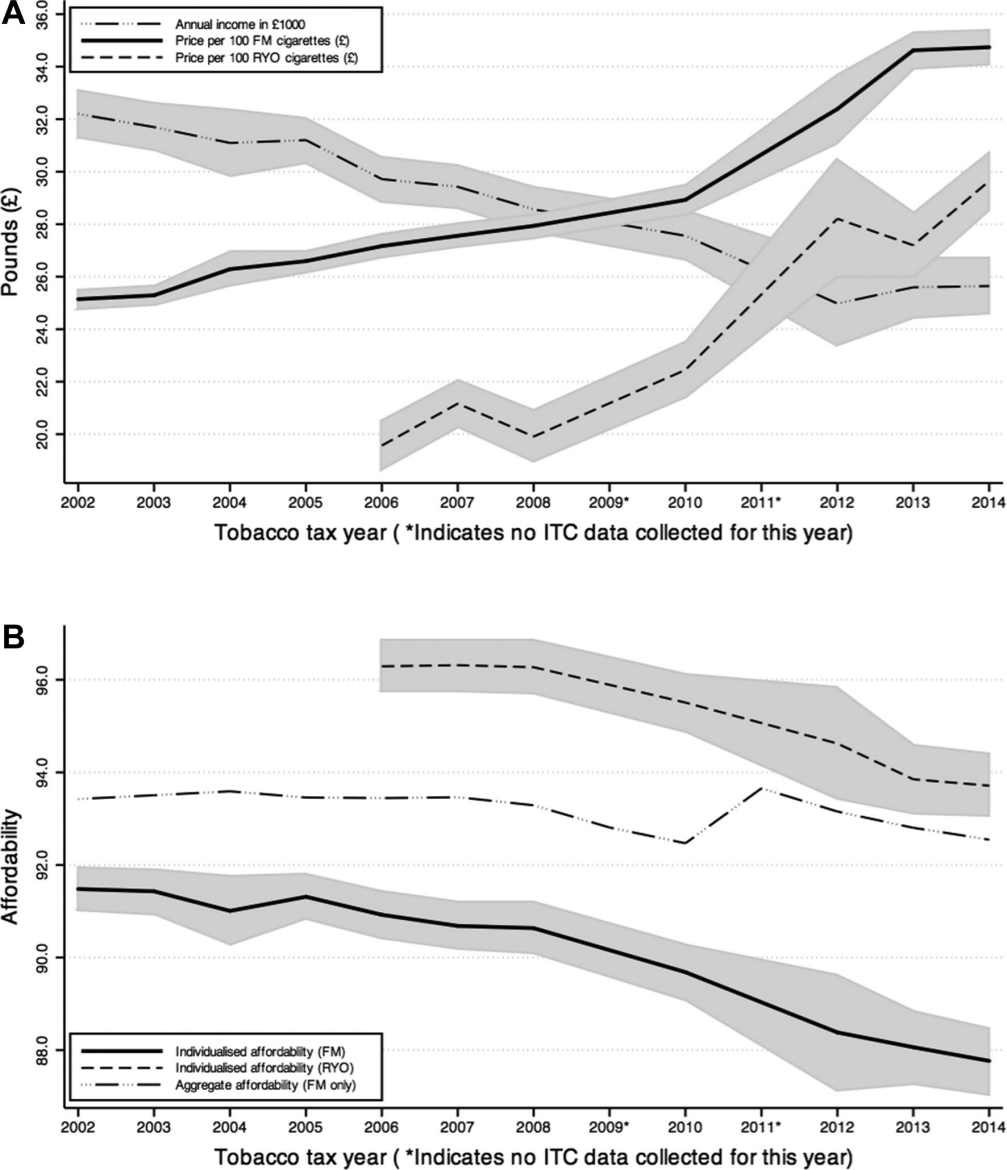
Measures of affordability, and their constituent components, over time, with shaded areas indicating 95% CIs. (A) indicates annual gross income (in £1000) and tobacco prices (per 100 cigarettes, in £) separately for factory made (FM) cigarettes and roll-your-own (RYO) tobacco. Values are adjusted for inflation using the consumer price index, with 2014 as the base year. (B) shows the individualised measures of affordability over time for FM cigarettes and RYO tobacco, and for the aggregate affordability measure (FM cigarettes only). Individualised affordability is unadjusted and inclusive of all sources (model 1, see text for details). Aggregate affordability is based on annual per capita gross domestic product and mean cigarette prices from annual sales in the most popular price category (prior to 2011) or the weighted average price (2011 onwards) for fully taxed UK sources only. ITC, International Tobacco Control.

An average annual increase in prices of 2.6% for FM cigarettes and 4.5% for RYO tobacco, and an average annual decrease of 1.6% in incomes (figure 1A) both contributed to a small decrease in individualised affordability (figure 1B) over the survey period. The income of the smokers in our sample deviated considerably from the national annual per capita GDP. Between 2002 and 2007, GDP increased from £26 206 to £30 299, whereas income for our sample *decreased* from £32 202 to £29423, and continued to decrease at a more marked rate than GDP to a low of £24 976 in 2012 (GDP reached a low of £27 196), after which both indicators saw a modest increase (see also the online supplementary figure S1). The affordability of FM cigarettes decreased at an average annual rate of 0.24%, from 91.5% (±95% CI: 91.0% to 91.9%) in 2002 to 87.8% (±95% CI: 87.0% to 88.5%) in 2014. The affordability of RYO tobacco decreased at an average annual rate of 0.31%, from 96.3% (±95% CI: 95.7% to 96.9%) in 2006 to 93.7% (±95% CI: 93.0% to 94.4%) in 2014. Affordability was significantly lower for FM cigarettes than RYO tobacco. These figures are unadjusted for any covariates and inclusive of all purchase sources (model 1). Aggregate affordability (fully taxed FM cigarettes only) also decreased. The spike that was evident between 2010 and 2011 coincides with the period during which the calculation of cigarette prices changed from the MPPC to the WAP, marking the switch between these two data series. Aggregate affordability decreased at an average annual rate of 0.13% between 2002 (93.4%) and 2010 (92.5%) when MPPC was used, and at an average annual rate of 0.40% between 2011 (93.7%) and 2014 (92.5%) when the WAP was used.

10.1136/tobaccocontrol-2017-054027.supp1 Supplementary data

For FM cigarettes (table 2), the unadjusted regression (model 1) indicated a significant linear trend, χ^2^(1)=118.8, p<0.0001, in the decrease in individualised affordability over time. Reverse adjacent contrasts, however, indicated that none of the year-on-year decreases were significant, with the exception of the two instances where there was a two-year interval between surveys, in 2008–2010, χ^2^(1)=8.9, p<0.005, and 2010–2012, χ^2^(1)=4.0, p<0.05. The same pattern of results was obtained for the fully adjusted model 2. When we excluded purchases made from non-UK/non-store sources (model 3), there was still a significant linear decrease in affordability with time, χ^2^(1)=54.3, p<0.0001. Adjacent contrasts, however, showed that none of the year-on-year decreases remained statistically significant. Model 3 for FM cigarettes is the most comparable to the aggregate affordability measure (inclusive only of FM cigarettes from fully taxed UK sources). Individualised affordability in model 3 was around 3%–6% lower than aggregate affordability each year, with a more marked decline. In both models 2 and 3 (adjusted for all covariates), 2010 was the first year that FM cigarettes became significantly less affordable than they had been in 2002. Changes in individualised affordability over time for RYO tobacco (Table 3) were similar to FM cigarettes. The unadjusted analysis (Model 1) indicated a significant linear trend, χ^2^(1) = 69.9, *p* < .0001, in the decrease of affordability over time, but only the decrease from 2008 to 2010, χ^2^(1) = 5.3, *p* < .05, was statistically significant in the reverse adjacent contrasts. The fully adjusted Model 2 also indicated a significant linear decrease over time, but no significant year-to-year changes. The same pattern was observed when purchases from non-UK/ non-store sources were excluded (Model 3).

**Table 2:** Linear random effects regression analyses of affordability regressed on time (tax year) and other covariates, for factory-made (FM) cigarette smokers only

	Model 1 n=3420, Obs=7475		Model 2 n=3420, Obs=7475		Model 3 n=3175, Obs=6439	
	β	SE	β	SE	β	SE
Time (tobacco tax year)						
2002	ref	---	ref	---	ref	---
2003	−0.05	0.23	−0.05	0.23	0.02	0.26
2004	−0.47	0.41	−0.44	0.40	−0.41	0.46
2005	−0.17	0.27	−0.07	0.27	−0.05	0.30
2006	−0.56	0.29	−0.36	0.28	−0.42	0.32
2007	−0.80**	0.30	−0.54	0.30	−0.60	0.34
2008	−0.84**	0.31	−0.38	0.31	−0.53	0.35
2010	−1.80***	0.35	−1.11**	0.35	−1.18**	0.40
2012	−3.10***	0.66	−2.45***	0.66	−2.57**	0.77
2013	−3.42***	0.44	−2.56***	0.44	−2.58***	0.48
2014	−3.71***	0.41	−2.82***	0.42	−3.10***	0.47
Time (tobacco tax year), reverse adjacent contrasts						
2003 vs 2002	−0.05	0.23	−0.05	0.23	0.02	0.27
2004 vs 2003	−0.42	0.42	−0.40	0.40	−0.43	0.46
2005 vs 2004	0.31	0.38	0.38	0.38	0.36	0.43
2006 vs 2005	−0.39	0.26	−0.29	0.26	−0.38	0.30
2007 vs 2006	−0.24	0.27	−0.18	0.27	−0.17	0.31
2008 vs 2007	−0.05	0.28	0.16	0.28	0.07	0.32
2010 vs 2008	−0.95**	0.32	−0.73*	0.31	−0.65	0.35
2012 vs 2010	−1.30*	0.66	−1.33*	0.65	−1.39	0.60
2013 vs 2012	−0.32	0.72	−0.11	0.71	−0.01	0.82
2014 vs 2013	−0.30	0.46	−0.25	0.45	−0.52	0.50
Sex						
Female	ref	---	ref	---	ref	---
Male	2.11***	0.34	1.93***	0.31	2.05***	0.34
Age (continuous)						
Age	0.22***	0.058	0.23***	0.05	0.23***	0.06
Age squared	−0.0036***	0.00059	−0.003***	0.00060	−0.003***	0.00060
Region						
London	ref	---	ref	---	ref	---
Yorkshire & The Humber	−1.00	0.69	−0.45	0.64	−0.49	0.70
East Midlands	−0.77	0.74	−0.46	0.69	−0.56	0.75
Eastern	−1.09	0.68	−0.29	0.64	−0.34	0.70
North East	−2.71**	0.86	−1.82*	0.81	−2.43**	0.89
South East	−0.42	0.59	0.19	0.55	0.35	0.60
South West	−1.84*	0.77	−0.71	0.72	−0.65	0.78
West Midlands	−1.57*	0.70	−0.82	0.65	−0.91	0.71
North West	−1.85**	0.65	−0.85	0.61	−0.96	0.66
Wales	−1.59	0.85	−0.33	0.80	−0.35	0.86
Scotland	−3.07***	0.65	−1.50*	0.61	−1.44*	0.65
Northern Ireland	−5.51***	1.00	−4.09***	0.93	−3.90***	0.97
Ethnicity						
White	ref	---	ref	---	ref	---
Not white	1.65*	0.72	−0.26	0.68	−0.22	0.73
Education						
Low	ref	---	ref	---	ref	---
Moderate	2.17**	0.36	1.45**	0.35	1.58***	0.38
High	5.14**	0.45	4.23**	0.44	4.48***	0.48
Time to first cigarette (TTFC)						
Over 60 min	ref	---	ref	---	ref	---
31 to 60 min	−1.25***	0.31	−1.19***	0.30	−1.23***	0.34
6 to 30 min	−2.33***	0.31	−2.21***	0.30	−2.39***	0.34
Within 5 min	−3.87***	0.38	−3.83***	0.37	−4.33***	0.41
Purchase source						
UK store-based	ref	---	ref	---	---	---
Non-UK/non-store	4.15***	0.25	4.10***	0.25	---	---

Note: model 1 is the unadjusted effects, of affordability regressed separately on each predictor variable, model 2 is adjusted for all covariates and model 3 is adjusted for all covariates but excluding purchases from non-UK/non-store sources. (table 3)

*P<0.05; **P<0.01; ***P<0.001.

**Table 3:** Linear random effects regression analyses of affordability regressed on time (tax year) and other covariates, for roll-your-own (RYO) tobacco smokers only

	Model 1 n=734, Obs=1468		Model 2 n=734, Obs=1468		Model 3 n=598, Obs=1056	
	β	SE	β	SE	β	SE
Time (tax year)						
2006	ref	---	ref	---	ref	---
2007	0.02	0.31	0.26	0.30	0.32	0.42
2008	−0.02	0.33	0.18	0.32	0.20	0.45
2010	−0.78*	0.35	−0.36	0.34	−0.35	0.46
2012	−1.68**	0.64	−0.92	0.63	−1.50	0.83
2013	−2.44***	0.42	−1.94***	0.41	−2.37***	0.53
2014	−2.58***	0.40	−1.80***	0.40	−2.16***	0.51
Time (tax year), reverse adjacent contrasts						
2007 vs 2006	0.02	0.31	0.26	0.30	0.32	0.42
2008 vs 2007	−0.04	0.32	−0.08	0.31	−0.12	0.42
2010 vs 2008	−0.76*	0.33	−0.54	0.33	−0.55	0.44
2012 vs 2010	−0.88	0.63	−0.57	0.62	−1.15	0.82
2013 vs 2012	−0.77	0.68	−1.01	0.67	−0.87	0.86
2014 vs 2013	0.14	0.42	0.13	0.41	0.21	0.50
Sex						
Female	ref	---	ref	---	ref	---
Male	0.64	0.41	0.90*	0.39	0.98*	0.48
Age (continuous)						
Age	0.16	0.087	0.16*	0.08	0.14	0.10
Age squared	−0.0024**	0.00088	−0.002**	0.00	−0.002*	0.00
Region						
London	ref	---	ref	---	ref	---
Yorkshire & The Humber	−0.87	1.02	−0.84	0.97	−0.98	1.18
East Midlands	−0.38	0.92	−0.64	0.88	−0.88	1.08
Eastern	0.49	0.87	0.67	0.83	0.89	1.03
North East	−0.84	1.18	−0.77	1.12	−0.91	1.32
South East	0.72	0.83	0.41	0.79	0.45	0.98
South West	0.64	0.83	0.57	0.79	0.79	0.95
West Midlands	−0.99	0.93	−0.82	0.88	−0.76	1.06
North West	1.14	0.91	0.89	0.86	0.74	1.04
Wales	−1.83	0.97	−1.94*	0.93	−2.63*	1.12
Scotland	−0.44	0.96	0.18	0.92	0.22	1.10
Northern Ireland	−1.15	1.39	−0.38	1.32	−0.02	1.49
Ethnicity						
White	ref	---	ref	---	ref	---
Not white	−0.89	1.19	−0.08	1.13	−0.22	1.30
Education						
Low	ref	---	ref	---	ref	---
Moderate	1.60***	0.45	1.49***	0.43	1.98***	0.52
High	2.01***	0.55	1.75**	0.54	2.26**	0.66
Time to first cigarette (TTFC)						
Over 60 min	ref	---	ref	---	ref	---
31 to 60 mins	0.03	0.51	−0.05	0.48	−0.18	0.62
6 to 30 min	−0.81	0.49	−1.10*	0.40	−1.42*	0.60
Within 5 min	−1.68**	0.56	−2.00***	0.53	−2.79***	0.69
Purchase source						
UK store-based	ref	---	ref	---	---	---
Non-UK/non-store	2.50***	0.31	2.35***		---	---

*Note:* model 1 is the unadjusted effects of affordability regressed separately on each predictor variable, model 2 is adjusted for all covariates and model 3 is adjusted for all covariates but excluding purchases from non-UK/nonstore sources.

*P<0.05; **P<0.01; ***P<0.001.

### Individual differences in affordability

**Figure 2:**
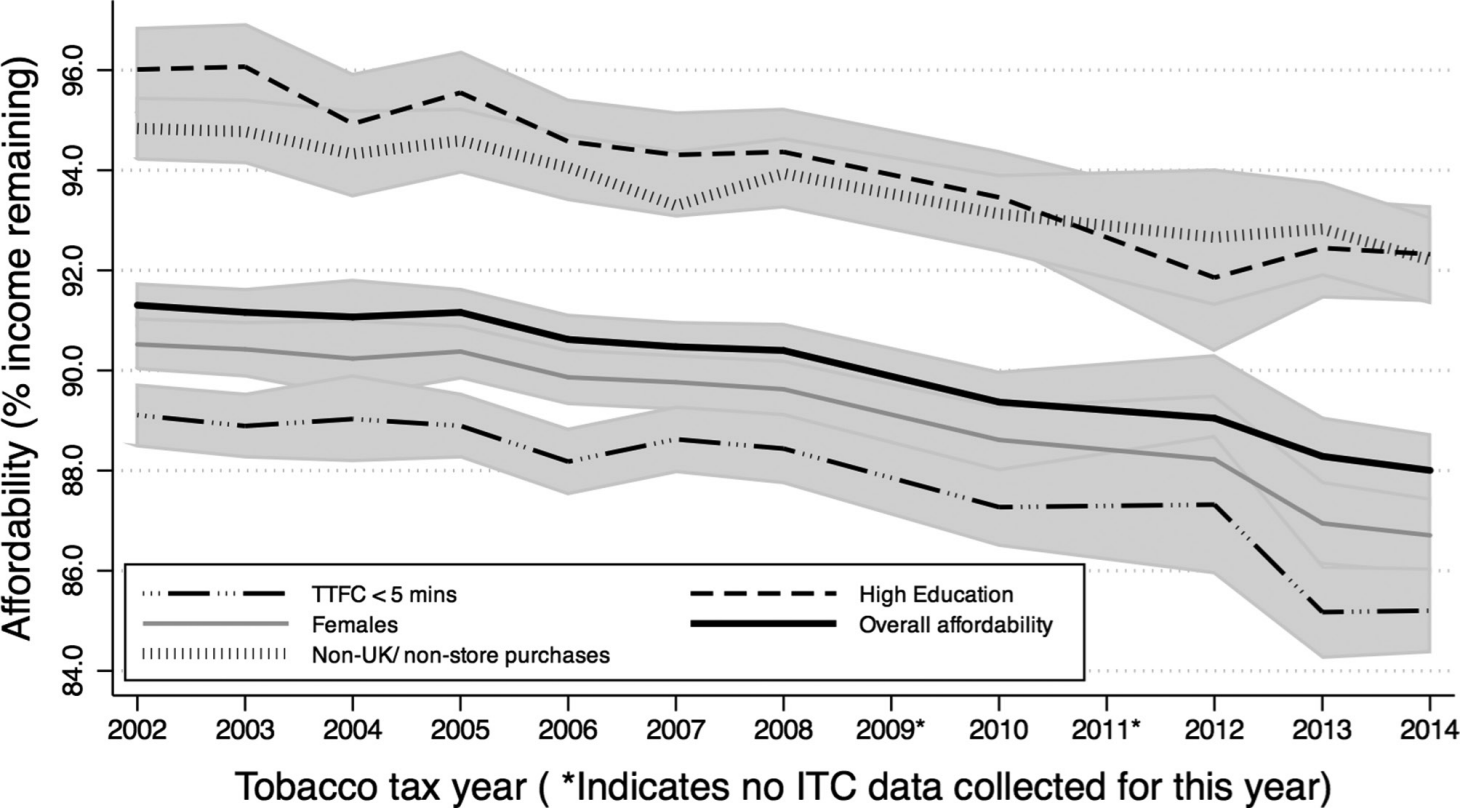
Individualised affordability for female smokers, those with high education, those who smoke their first cigarette within 5 min of waking (TTFC < 5 min), and those purchasing from non-UK/nonstore sources, compared with overall affordability. Shaded areas represent 95% CI. Affordability estimates are from the fully adjusted model 2 for FM smokers only, and adjusted for covariates (see text for details). FM, factory-made; ITC, International Tobacco Control; TTFC, time to first cigarette.

Unadjusted regressions (model 1) indicated that FM cigarettes were significantly less affordable for females, smokers from the North East and western regions of England, Scotland and Northern Ireland (compared with London), white smokers, those with the lowest level of education, more dependent smokers and those who purchased cigarettes from UK store-based sources (table 2). There was also a significant inverse quadratic association between affordability and age, such that smoking was most affordable for smokers aged around 36 years, somewhat less affordable for younger smokers and much less affordable for the oldest smokers. Only minor changes were observed to this pattern of associations in the fully adjusted model 2 (western regions of England no longer differed significantly from London, and differences by ethnicity were no longer significant), and no further changes were observed when we excluded purchases from non-UK/non-store sources in model 3. figure 2 presents individualised affordability of FM cigarettes over time for different demographic groups, where it is evident that large savings can be made by purchasing from non-UK/nonstore sources, and that the most dependent smokers are spending relatively more of their income on tobacco.

For RYO smokers (table 3), the unadjusted model 1 indicated significantly less affordable tobacco for those with the lowest level of education, those who smoked their first cigarette within 5 min of waking (versus after 60 min) and smokers who purchased from UK store-based sources. In the fully adjusted model 2, the inverse quadratic association between age and affordability that was observed for FM cigarettes became statistically significant, and RYO tobacco was also significantly less affordable for smokers from Wales (versus London). This pattern of associations persisted when we excluded purchases from non-UK/non-store sources in model 3, with the exception that the association with age again became non-significant.

### Sensitivity analysis with tobacco consumption (CPD) as the outcome

The sensitivity analysis indicated a small but significant reduction in cigarette consumption (CPD) over time among FM smokers, from 17.5 (95% CI: 17.2 to 17.9) in 2002 to 16.1 (95% CI: 15.6 to 16.7) in 2014, with a significant linear trend χ^2^(1)=40.9, *P*<.0001. No significant change in CPD over time was observed for RYO smokers, from 16.8 (95% CI: 16.0 to 17.6) in 2006 to 17.3 (95% CI: 16.4 to 18.3) in 2014, and the linear trend was not statistically significant χ^2^(1)=1.7, *P*=.20. The observed decreases in affordability were thus not attributable to changes in cigarette consumption.

## Discussion

Our new individualised measure of affordability indicated that tobacco in the UK was significantly less affordable in 2014 than it had been in 2002. Smokers of FM cigarettes retained 91.5% of their income after paying for tobacco in 2002, but only 87.8% in 2014. For the first time, we have been able to assess affordability for RYO smokers and we found that tobacco was more affordable for RYO smokers, but this too decreased significantly, from 96.3% in 2006 to 93.7% in 2014. The decrease was not attributable to changes in cigarette consumption, but to decreases in income of our sample (1.6% annually) and increases in mean cigarette prices (2.6% and 4.5% annually for FM and RYO, respectively), both contributing to the decrease in affordability. Our analyses highlighted an advantage of our new individualised measure of affordability, in that it is able to capture income endogeneity. The average incomes of our sample of smokers deviated considerably from national averages based on annual per capita GDP. As smoking is increasingly becoming associated with widening socioeconomic disparities, this is particularly important, and affordability measures using estimates of income based on national averages are less able to capture these shifts.

The decrease in affordability, however, was much lower (0.24% and 0.31% annually for FM and RYO, respectively) than what would be expected from the observed changes in income and cigarette prices. This suggests that individual characteristics play a role. Indeed, tobacco was least affordable for female smokers, older smokers, those with low levels of education and more highly dependent smokers. It also indicates that smokers are able to manage tobacco affordability through their purchase patterns. Smoking RYO tobacco instead of FM cigarettes saved up to 5% of smokers' annual incomes (around £1300 in 2014). Purchasing from overseas, duty free or informal/ illicit sources also conferred a saving of around 5%.

Despite the overall decrease in affordability between 2002 and 2014, the year-on-year changes were not statistically significant (except when there were two-year instead of one-year gaps between surveys) and thus probably not substantial enough to prompt smokers, especially more dependent smokers, to quit. The period between 2008 and 2012 saw the sharpest rate of decrease in affordability (see figure 1B). It was only from 2009 that UK tobacco taxes were greater than the rate of inflation during the study period,^23^ and an especially large increase in RYO tobacco taxes occurred in 2011,^25^ when the sharpest increase in RYO prices was observed (figure 1A). This finding clearly underscores the importance of large tax increases that result in tobacco price increases greater than the rate of inflation and for measures that differentially increase RYO taxes in order to reduce the price gap between FM and RYO tobacco products. Indeed, it was only from 2010 onwards that FM cigarettes became significantly less affordable than they had been in 2002. The sharpest drop in incomes for our sample also occurred from 2010 onwards, likely a result of the 2008-09 economic recession. Unfortunately, due to the overlapping timeframes of these changes and gaps in our data collection (no surveys in 2009 or 2011), we cannot conclusively determine the strength of their relative contributions to the changes in affordability. Nevertheless, our findings support the need for large tax increases above the rate of inflation and taxing all tobacco products in a way that minimises the incentive to substitute with cheaper products.

A steady decline in incomes was observed among our sample from 2002 to 2012, yet the wealth of the UK population as a whole was increasing prior to the recession in 2008.^30^ This supports the theory that smoking is increasingly becoming a hallmark of socioeconomic disadvantage.^2 32 33^ Indeed, some of the observed individual differences in affordability, such as lower affordability for females, very old smokers, those of low education and regional variations, can plausibly be attributed to lower incomes among these groups. The most dependent smokers are spending about 2% (RYO) to 4% (FM) more of their incomes (around £500 to £1000 annually in 2014) on tobacco than the least dependent. Providing additional support to these smokers, for whom it is hardest to quit,^32 34^ must remain a priority for policy-makers. In addition, policy-makers should ensure that taxes are applied differentially according to risk: for example, less harmful nicotine products such as nicotine replacement therapies and electronic cigarettes should be taxed at levels commensurate with their relative risks comparable to smoking.^35^

We compared our individualised affordability measure to an aggregate version based on average cigarette prices and national estimates of income. The aggregate measure gave estimates of affordability for FM cigarettes that were about 3%–6% higher each year than our individualised measure, and the decline over time was also less marked for aggregate affordability. We believe that our individualised measure offers some benefits over and above the aggregate measure, like the measures of tobacco affordability that are currently in use.^5 8–10^ These are that it takes individual variations in consumption into account, considers untaxed or illicit purchase sources and the use of RYO tobacco. It is therefore likely that our individualised measure more accurately reflects actual changes in tobacco affordability over time than do aggregate measures, and where feasible, can provide a complementary measure to the extant aggregate affordability measures.

### Limitations

Our sample only included current smokers. Some recent ex-smokers may have quit due to their low levels of tobacco affordability. If this were the case, our data would somewhat underestimate the decrease in affordability. Future research using our individualised measure can explore this by comparing affordability among recent quitters to continuing smokers. Due to insufficient data, nondaily smokers and those who habitually smoked both FM cigarettes and RYO tobacco were also excluded. This may have slightly underestimated affordability, as these groups might be particularly adept at controlling their tobacco expenditure, smoking less or switching between FM and RYO as needed. Our analysis used repeat cross-sectional data; future studies could assess within subject changes in the individualised affordability measure over time. Future research in this way would also help establish whether differential quitting across different socioeconomic groups is contributing to the decreases in income observed overall in our sample of smokers. Lastly, we only had data on gross (before tax) rather than net (after tax) income. Individuals in the UK with higher incomes are taxed at progressively higher rates, so their net income (what is actually available to spend on tobacco) will be reduced by relatively more than those on lower incomes. For the high-income groups, affordability will therefore be slightly overestimated. However, we do not expect this to cause a large bias in our estimates, as the majority of our sample (68%) had gross incomes below £30  000, which was below the threshold for moving beyond the lowest tax rate in all the years analysed, with the exception of the 2002–2003 tax year where the threshold was £29  000.^36^ Future studies, however, might improve on our methodology by using net instead of gross income to calculate affordability. Our measure of affordability relied on high-quality ITC data, and the growing number of countries participating in the ITC increase its applicability.

## Conclusions

The newly developed individualised measure of tobacco affordability complements aggregate measures based on national estimates of income and average tobacco prices by providing a more accurate and nuanced insight into the impact of tobacco taxes. Tobacco in the UK was significantly less affordable in 2014 than in 2002, although the rate of decrease was low (0.24% annually) and year-on-year declines were not significant. Affordability was modified by larger tax increases, in addition to social inequalities and purchase patterns. More dependent smokers and those of low socioeconomic status spent relatively more of their incomes on tobacco. RYO tobacco was considerably more affordable than FM cigarettes, and policy-makers need to focus on closing this gap.

**What this paper adds**

**What is already known on this subject**

- Raising cigarette prices through tobacco taxation is an effective tobacco control measure, but it is impacted by inflation rates and changes in income. Affordability measures have been developed to enable these considerations to be taken into account and such measures have been standardised to enable comparisons over time and across countries. Extant measures use national income estimates and average cigarette prices of Marlboro or local brands.

**What important gaps in knowledge exist on this topic**

- Aggregate affordability measures can be problematic given wide income inequalities and the range of prices across brands and different types of tobacco (such as factory made (FM) and roll-your-own (RYO). In addition they cannot account for smokers ‘strategies to minimise costs, such as buying in bulk or from cheaper sources.

**What this paper adds**

- This study developed a complementary individualised measure of tobacco affordability using smokers' own incomes and FM and (for the first time) RYO tobacco purchase prices. Both FM and RYO became less affordable over time, but RYO was significantly more affordable than FM and the annual rate of decline in individualised affordability was low, suggesting annual tax rises were not large enough. Individual characteristics and purchasing decisions influenced tobacco affordability.
