# Supplementary figures and images for: Individualised tobacco affordability in the UK 2002–2014: findings from the International Tobacco Control Policy Evaluation Project
Source: Tob Control. 2018 Jul 23;28(Suppl 1):s9–s19. doi: 10.1136/tobaccocontrol-2017-054027 (PMC6580872; doi:10.1136/tobaccocontrol-2017-054027)

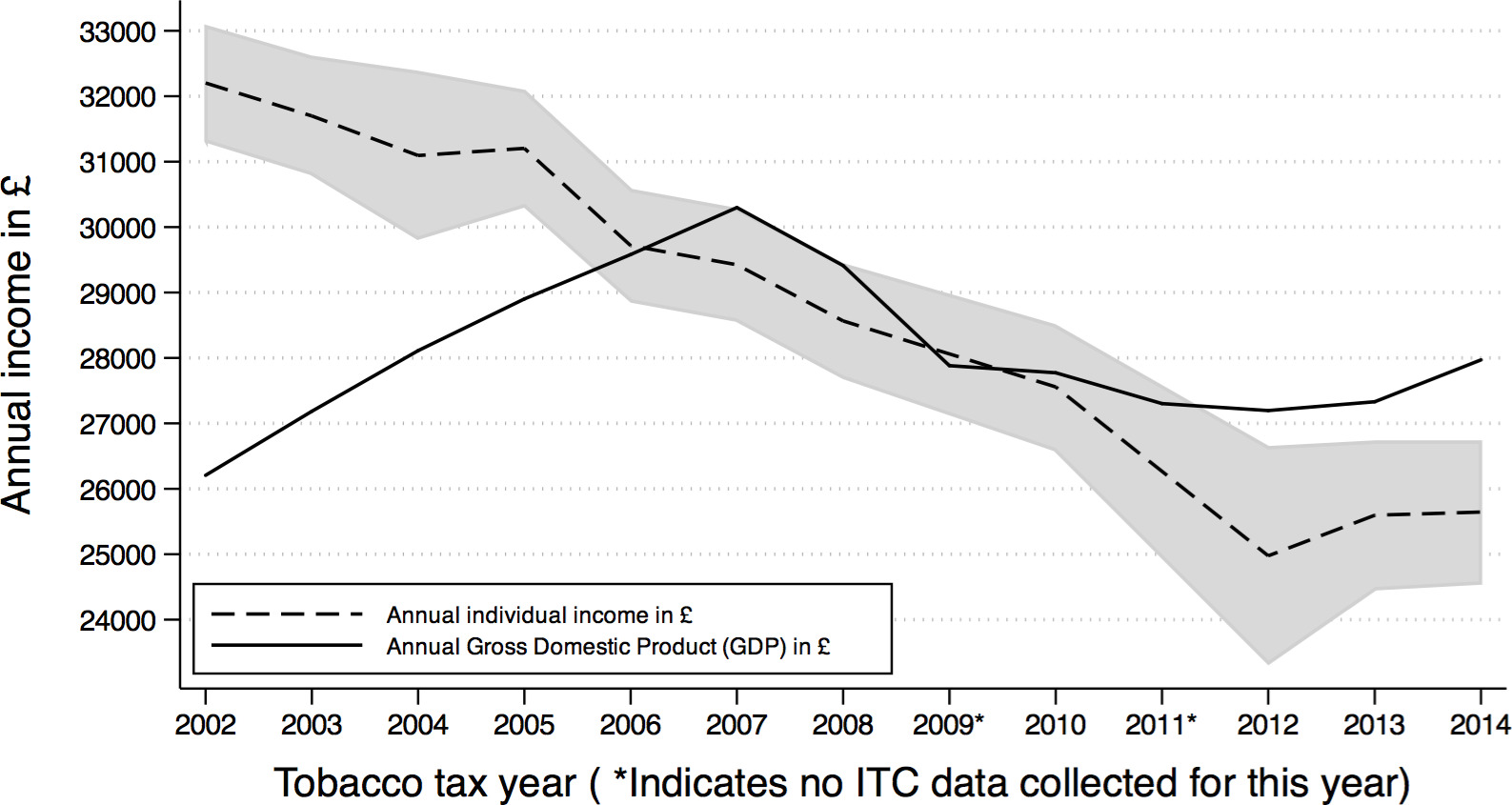

Supplement: Supplementary data [file tobaccocontrol-2017-054027supp001.jpg]
